# Chronic Omega-3 Polyunsaturated Fatty Acid Treatment Variably Affects Cellular Repolarization in a Healed Post-MI Arrhythmia Model

**DOI:** 10.3389/fphys.2016.00225

**Published:** 2016-06-14

**Authors:** Ingrid M. Bonilla, Yoshinori Nishijima, Pedro Vargas-Pinto, Stephen H. Baine, Arun Sridhar, Chun Li, George E. Billman, Cynthia A. Carnes

**Affiliations:** ^1^College of Pharmacy, The Ohio State UniversityColumbus, OH, USA; ^2^Department of Physiology and Cell Biology, The Ohio State UniversityColumbus, OH, USA; ^3^Dorothy M. Davis Heart and Lung Research Institute, The Ohio State UniversityColumbus, OH, USA; ^4^Department of Veterinary Biosciences, College of Veterinary Medicine, The Ohio State UniversityColumbus, OH, USA; ^5^Division of Cardiology, Peking University People's HospitalBeijing, China

**Keywords:** omega-3 polyunsaturated fatty acids, ventricular fibrillation, electrophysiology, potassium current, myocardial infarction

## Abstract

**Introduction:** Over the last 40 years omega-3 polyunsaturated fatty acids (PUFAs) have been shown to be anti-arrhythmic or pro-arrhythmic depending on the method and duration of administration and model studied. We previously reported that omega-3 PUFAs do not confer anti-arrhythmic properties and are pro-arrhythmic in canine model of sudden cardiac death (SCD). Here, we evaluated the effects of chronic omega-3 PUFA treatment in post-MI animals susceptible (VF+) or resistant (VF−) to ventricular tachyarrhythmias.

**Methods:** Perforated patch clamp techniques were used to measure cardiomyocyte action potential durations (APD) at 50 and 90% repolarization and short term variability of repolarization. The early repolarizing transient outward potassium current I_to_ was also studied.

**Results:** Omega-3 PUFAs prolonged the action potential in VF− myocytes at both 50 and 90% repolarization. Short term variability of repolarization was increased in both untreated and treated VF− myocytes vs. controls. I_to_ was unaffected by omega-3 PUFA treatment. Omega-3 PUFA treatment attenuated the action potential prolongation in VF+ myocytes, but did not return repolarization to control values.

**Conclusions:** Omega-3 PUFAs do not confer anti-arrhythmic properties in the setting of healed myocardial infarction in a canine model of SCD. In canines previously resistant to ventricular fibrillation (VF−), omega-3 PUFA treatment prolonged the action potential in VF− myocytes, and may contribute to pro-arrhythmic responses.

## Introduction

Sudden cardiac death (SCD) caused by ventricular tachy-arrhythmias is the leading cause of death in the United States and contributes to 15–20% of deaths worldwide (Hayashi et al., 2015). Myocardial infarction and resulting scar tissue result in structural and electrical remodeling leading to an arrhythmogenic substrate; at the cellular level repolarization abnormalities and calcium dysregulation occur which can contribute to ventricular tachyarrhythmia formation and SCD (Rubart and Zipes, 2005; Wagner et al., 2015). Treatments that normalize repolarization may be anti-arrhythmic in the setting of healed MI and decrease the susceptibility to SCD (Billman, 2006).

The effects of omega-3 polyunsaturated fatty acid (PUFA) consumption and cardiovascular health have been studied for more than 40 years (Dyerberg et al., 1978). While observational studies suggest a role for omega-3 PUFAs in preventing cardiac mortality(Kromhout et al., 1985), results from studies using omega-3 PUFAs as secondary prevention of cardiovascular events have shown mixed results (Gillum et al., 1996; Erkkila et al., 2003; Wilhelm et al., 2008; Smith et al., 2009). Despite mixed results of prior studies the American Heart Association recommends eating fatty fish containing the active ingredients docosahexaenoic acid (DHA) and eicosapentaenoic acid (EPA) two times per week in patients with or without existing coronary heart disease (CHD) and 1 g of EPA +DHA by capsule per day in patients with existing CHD (Gebauer et al., 2006).

Cellular experiments have demonstrated that acute administration of omega-3 PUFAs are able to modulate a number ion currents including calcium, sodium, and potassium currents (Dhein et al., 2005; Guizy et al., 2005; Meves, 2008). However, it is not clear if these effects are pro-arrhythmic or anti-arrhythmic (Belevych et al., 2013). We previously reported that acute administration of omega-3 PUFAs through intravenous infusion of a fish oil emulsion prevented ventricular fibrillation in canines susceptible to induction of ventricular fibrillation (VF+) in a clinically relevant model of SCD (Leaf et al., 2003). In contrast, dietary administration of omega-3 PUFAs did not confer protection against VF (Billman et al., 2012). Furthermore, omega-3 PUFAs were shown to be pro-arrhythmic and cause SCD in the same animal model (Billman et al., 2012). Additionally, Coronel et al. reported that chronic dietary administration of omega-3 PUFAs were pro-arrhythmic and shortened the action potential in a regionally ischemic porcine model (Coronel et al., 2007).

The purpose of the current study was to elucidate the cellular electrophysiological effects caused by chronic dietary omega-3 PUFAs supplementation in a post-MI canine model of sudden death at both high (VF+) and low (VF−) risk of developing ventricular arrhythmias.

## Materials and methods

All animal procedures were approved by The Ohio State University Institutional Animal Care and Use committee and conformed to *the Guide for the Care and Use of Laboratory Animals* published by the US National Institute of Health (NIH publication No. 85-23, revised 1996). Forty-three heartworm free mixed breed dogs had a left ventricular anterior myocardial infarction induced as previously described (Billman, 2006). Seventeen healthy age-matched dogs served as controls.

### *In vivo* preparation

The animal model, omega-3 PUFA treatment protocol, and *in vivo* assessments have been previously described (Sridhar et al., 2008; Billman et al., 2012). Briefly, the left anterior descending coronary artery was occluded to induce a left anterior myocardial infarction (Billman et al., 1982; Billman, 2006). After recovery (3–4 weeks) the susceptibility to ventricular fibrillation was tested using a standardized exercise plus ischemia test (Billman, 2006; Babu et al., 2007; Sridhar et al., 2008). The exercise plus ischemia test reliably induced ventricular flutter/fibrillation in animals which were then classified as VF+. Those without induced sustained ventricular tachy-arrhythmias were classified as VF− (Sridhar et al., 2008).

### Omega-3 PUFA protocol

**Figure 1:**
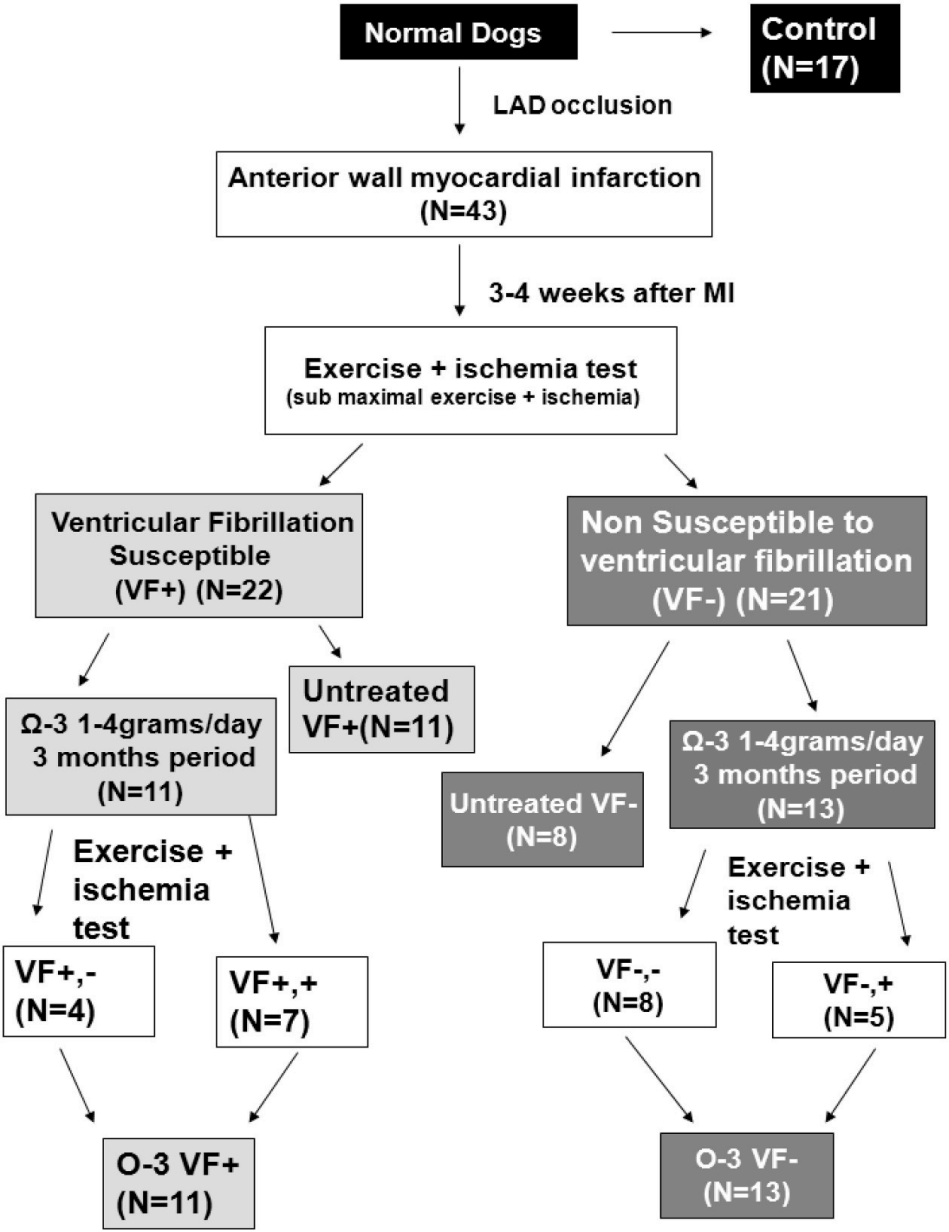
**Study design and treatment groups**. Three to four weeks after myocardial infarction, dogs were classified as susceptible or resistant to ventricular fibrillation (VF) with an exercise plus ischemia test. Three month treatment was randomly assigned, and the exercise plus ischemia test was repeated at the end of treatment. *N*, number of animals; LAD, Left anterior descending artery; MI, myocardial infarction.

To prevent confounding dietary omega-3 PUFA intake, dogs used for the omega-3 PUFA experiments were fed a diet free of omega-3 PUFAs (Harland Teklad, Harlan Laboratories, Inc., Indianapolis, IN, USA) for the duration of the study (~4 months; Billman et al., 2010, 2012). Dogs were randomly assigned to the following groups: untreated VF+ *n* = 11, untreated VF− *n* = 8, treated VF+ *n* = 11, and treated VF− *n* = 13 (Figure 1). Dogs in the treated group received 465 mg ethyl eicosapentaenoate, EPA + ethyl docosahexaenoate, DHA, 375 mg per 1 g capsule (Lovaza®, GlaxoSmithKline, Research Triangle Park, NC); doses of 1, 2, and 4 g where administered. The capsules were given orally prior to the daily feeding (7 days per week for 3 months) as previously reported (Billman et al., 2012). The exercise plus ischemia test was repeated after the omega-3 PUFA treatment period (Billman et al., 2012). Since no dose-dependent differences in response were found in this or previous work in this model (Billman et al., 2010), results from all dose groups were combined. Omega-3 PUFA treatment induced malignant arrhythmias in ~33% of the VF− dogs, indicating a pro-arrhythmic effect of treatment (Billman et al., 2012). In the VF+ dogs, omega-3 PUFA treatment did not confer any protection against malignant arrhythmias, as we previously reported (Billman et al., 2012). Since no significant cellular electrophysiological differences based on treatment response were found in either the VF− dogs or the VF+ dogs, canines were grouped as omega-3 PUFA treated VF− or VF+, respectively. Figure 1 provides a detailed schematic of the dog classification.

### Myocyte isolation

Myocytes were isolated as previously described (Sridhar et al., 2008; Bonilla et al., 2014). Briefly, left ventricular myocytes were isolated a minimum of 5 days after the final exercise plus ischemia test to avoid potential residual effects of acute ischemia. Dogs where anesthetized by intravenous injection of pentobarbital sodium (dosage: 120 mg/kg for the first 4.5 kg and 60 mg/kg for every 4.5 thereafter). After a deep plane of anesthesia was achieved, the heart was rapidly removed and perfused with cold cardioplegia solution containing the following in mM: NaCl 110, CaCl_2_ 1.2, KCl 16, MgCl_2_ 16, and NaHCO_3_ 10. Cannulation of the left circumflex artery was used to perfuse the left ventricle as previously described (Bonilla et al., 2012). In post-MI dogs a clear margin of infarct was visible as scar tissue. After enzymatic digestion of the left ventricle (Bonilla et al., 2012; Belevych et al., 2013), the digested non-infarcted mid myocardial section (at least 1 cm from the scar border) was used as the source of myocytes. This method typically yielded 50–70% rod shaped ventricular myocytes. The myocytes were stored at room temperature in a standard incubation buffer containing the following in mM: 118 NaCl, 4.8 KCl, 1.2 MgCl_2_, 1.2 KH_2_PO_4_, 0.68 glutamine, 10 glucose, 5 pyruvate, 1 CaCl_2_, along with 1 μmol/L insulin, and 1% BSA until use. All myocyte electrophysiological experiments were conducted within 10 h of myocyte isolation.

### Electrophysiological protocols

To assess myocyte electrophysiology amphotericin-B perforated patch clamp techniques with a bath temperature of 36 ± 0.5°C were used. Myocytes were placed in a laminin coated cell chamber (Cell Microcontrols, Norfolk, VA) and superfused with bath solution containing in mM: 135 NaCl, 5 MgCl_2_, 5 KCl, 10 glucose, 1.8 CaCl_2_, and 5 HEPES with pH adjusted to 7.40 with NaOH. Borosilicate glass micropipettes with tip resistance of 1.5–3 MΩ were filled with pipette solution containing the following in mM: 100 K-aspartate, 40 KCl, 5 MgCl, 5 EGTA, 5 HEPES, pH adjusted to 7.2 with KOH.

Action potential duration (APD) measurements were obtained from the average of the last 10 traces (steady state) from a train of 25 action potentials elicited at each stimulation rate. To evaluate beat to beat variability, Poincaré plots of the last 10 consecutive beats were drawn by plotting each APD90 (APD90 *n* + 1) against the APD90 of the previous beat (APD90 *n*) as previously reported (Varkevisser et al., 2012; Belevych et al., 2013). Short term variability of APD90, expressed in ms, was calculated by using the following formula: $\sum \left(APD90n+1-APD90n\right)∕\left(10\times \sqrt{2}\right)$ as previously reported (Varkevisser et al., 2012; Belevych et al., 2013).

For I_to_ recordings, only recordings with an access resistance < 20 MΩ were included in the analyses. Calcium in the bath solution was reduced to 1.0 mM and 2 μM nifidepine was added to block L-type Ca^2+^ current. I_to_ was elicited from a holding potential of −60 mV by a series of 100 ms test potentials from −30 to +50 mV and measured as peak current minus steady state current as previously described (Belevych et al., 2013).

### Solutions and chemicals

All chemicals used for buffer and solution preparation were purchased from Sigma Aldrich (St. Louis, CS) unless otherwise noted. Cardioplegia and solutions containing nifidepine and amphotericin-B were prepared daily.

### Interstitial fibrosis

Transmural right ventricular tissue samples were formalin fixed and embedded in paraffin and sectioned to 5 μm thickness, using standard procedures. Tissue sections were stained with Masson's Trichrome to define the percentage area of fibrosis, as previously described (Nishijima et al., 2007).

### Data analysis

Cellular electrophysiology data were analyzed using Clampfit 10.3 software (Axon Instruments) and Origin 9.0 software (OriginLab, Northampton, MA, USA). Comparisons between three or more groups were analyzed by one-way ANOVA with *post hoc* least significant difference testing (Originpro 8.6, OriginLab). All data are presented as mean ± SE and *p* < 0.05 was the criterion for statistical significance for all comparisons.

## Results

### Omega-3 PUFA treatment of VF− canines causes a prolongation of cellular repolarization

**Figure 2:**
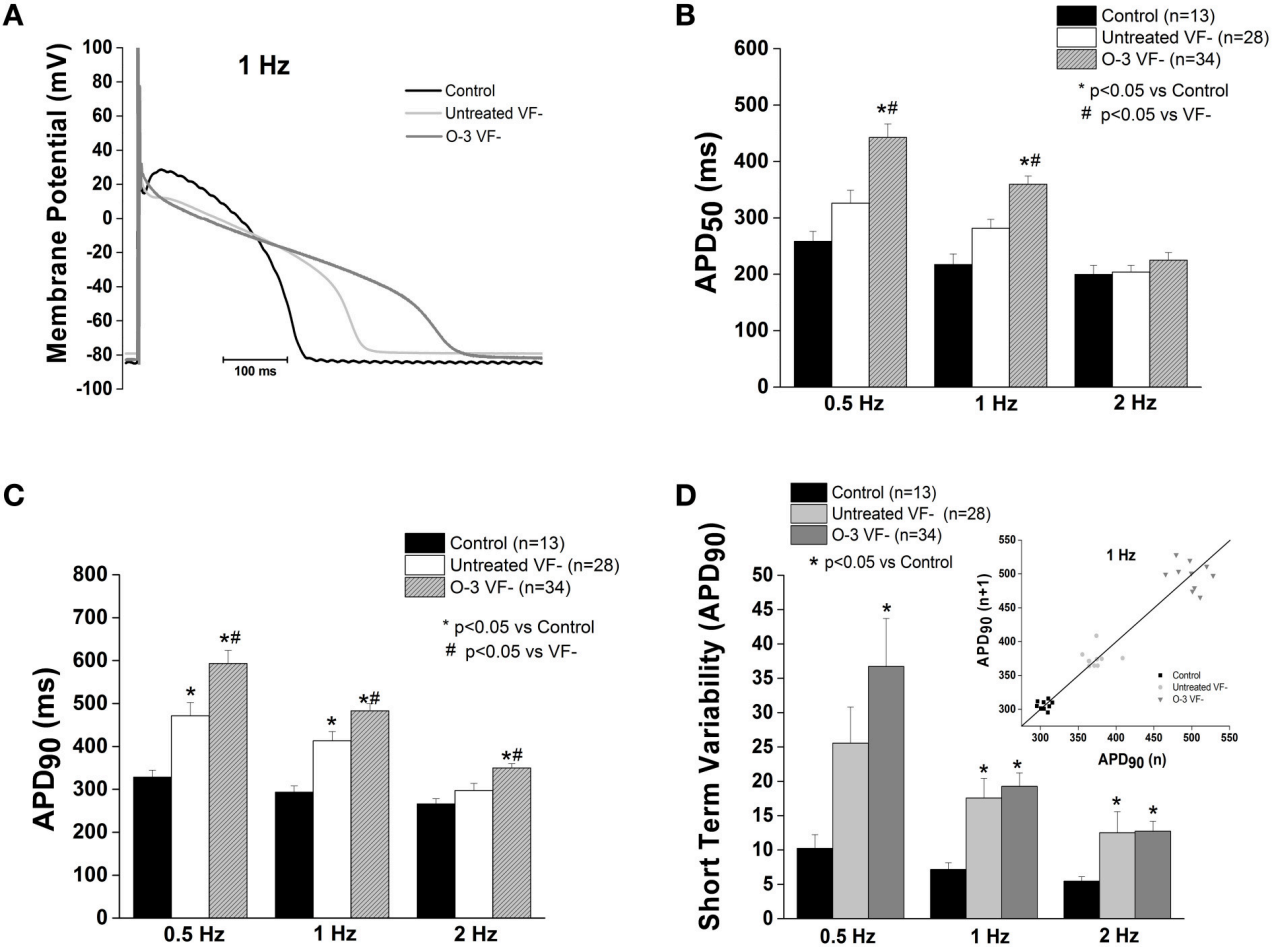
**Omega-3 PUFA treatment prolongs repolarization in VF− myocytes. (A)** Representative tracings of action potentials of control, untreated VF−, and omega-3 PUFA treated VF− cardiomyocytes. **(B)** APD_50_ is significantly increased in the omega-3 PUFA treated VF− myocytes compared to both control and untreated VF− cardiomyocytes (*P* < 0.05 vs. control and untreated VF− 0.5 and 1 Hz). **(C)** APD_90_ is significantly increased in the omega-3 PUFA treated group at all rates; compared to both control and untreated VF− cardiomyocytes (*P* < 0.05 vs. control and untreated VF− at 0.5, 1, and 2 Hz). **(D)** Short term variability of APD_90_ is not further increased by omega-3 PUFA treatment (*P* < 0.05 vs. control at all rates). Insert: Representative Poincaré Plot of APD_90_ from control, Untreated VF− and O-3 VF− groups, 6–8 animals per group, *n* = number of cells.

APD at 90% repolarization (APD90) in untreated VF− cells was significantly prolonged compared to control at 0.5 and 2 Hz (*p* < 0.05); no differences were observed in APD at 50% repolarization (APD50; Figure 2). However, omega-3 PUFA treatment of VF− dogs caused a significant prolongation of APD50 at 0.5 and 1 Hz (*p* < 0.05 vs. control and untreated VF−) and increased APD90 at all rates compared to untreated VF− (*p* < 0.05 vs. untreated VF− and control; Figure 2). Short term variability was measured to assess the instability of the action potential, a measure of pro-arrhythmic potential. Both untreated VF− and the omega-3 PUFA treated VF− groups had increased short term variability vs. control (*p* < 0.05), while omega-3 PUFA treatment did not affect short term variability (Figure 2).

### Cardiac transient outward potassium current (I_to_) is unaffected by Omega-3 PUFA treatment in VF− animals

**Figure 3:**
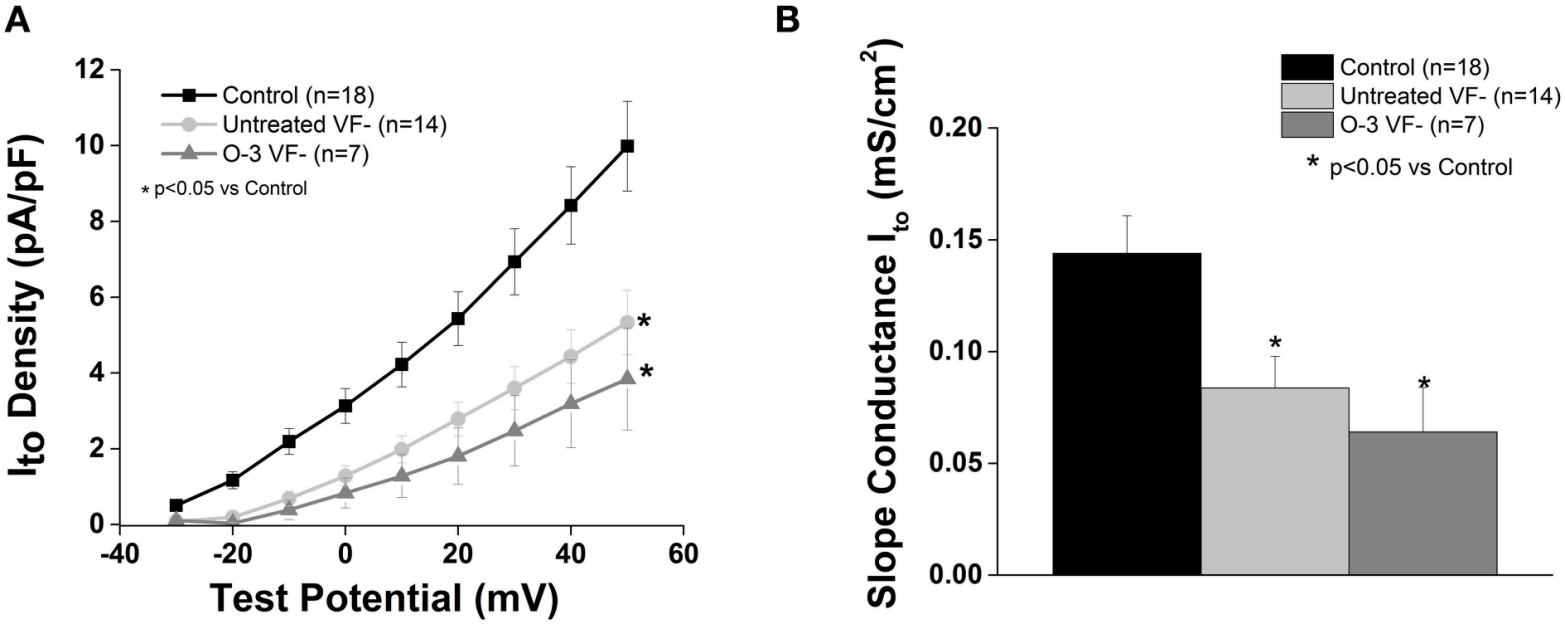
**Omega-3 PUFA treatment does not affect I_to_ in myocytes from VF− animals. (A)** I–V curve. **(B)** Slope conductance. I_to_ density and slope conductance is significantly lower in VF− vs. control cardiomyocytes and unaffected by omega-3 PUFA treatment (*P* < 0.05 vs. control). Three to six animals per group; *n* = number of cells.

In accordance with our previous study, I_to_ current was significantly reduced in the untreated VF− group compared to control (*p* < 0.05 vs. control). Omega-3 PUFA treatment had no effect on I_to_ current vs. untreated VF− canines (Figure 3).

### Omega-3 PUFA treatment of animals susceptible to ventricular arrhythmias (VF+) causes a shortening of the action potential

**Figure 4:**
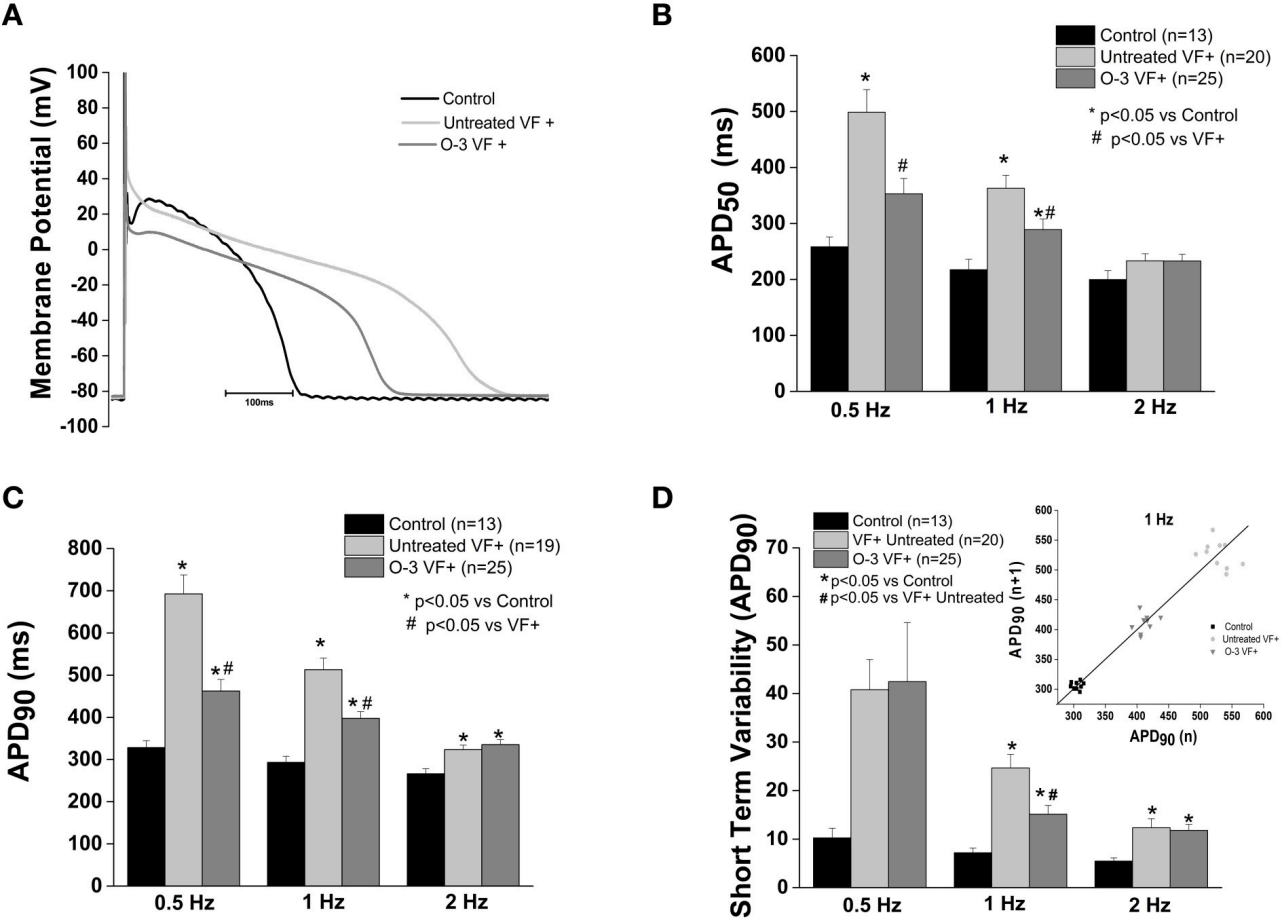
**Omega-3 PUFA treatment attenuates action potential duration prolongation in myocytes from VF+ myocytes**. **(A)** Representative tracings of action potentials of control, untreated VF+ and omega-3 PUFA treated VF+ cardiomyocytes. **(B)** APD_50_ is prolonged in VF+ myocytes at 0.5 and 1 Hz, the prolongation is attenuated only at 1 Hz (*P* > 0.05). **(C)** APD_90_ is significantly prolonged in the VF+ group; omega-3 PUFA treatment attenuated the prolongation at 0.5 and 1 Hz (*P* < 0.05). **(D)** Short term variability of APD_90_ is decreased only at 1 Hz, but remains significantly higher than in control cardiomyocytes (*P* < 0.05 vs. control at 1 Hz). Insert shows a representative Poincaré Plot of APD_90_ at 1 Hz from control, Untreated VF+ and O-3 VF+ groups. Five to seven animals per group; *n* = number of cells.

Previously we found that myocytes from animals susceptible to ventricular fibrillation (VF+) had a significantly prolonged APD50 and APD90 compared to controls (Sridhar et al., 2008). Omega-3 PUFA treatment caused a significant shortening of the APD50 (1 Hz) and APD90 (0.5 and 1 Hz) compared to untreated VF+, however, repolarization remained prolonged vs. control (*p* < 0.05 vs. control and untreated VF+). At 2 Hz omega-3 PUFA treatment did not alter APD (Figure 4).

Short term variability of the APD at 90% of repolarization (STV) in VF+ myocytes was significantly increased compared to control myocytes at 1 and 2 Hz (*P* < 0.05 vs. control). Omega-3 PUFA treatment caused a significant reduction of STV at 1 Hz, yet was increased relative to controls (*p* < 0.05 vs. control and untreated VF+, Figure 4).

### Cardiac transient outward potassium current (I_to_) is unaffected by Omega-3 PUFA treatment in VF+ animals

**Figure 5:**
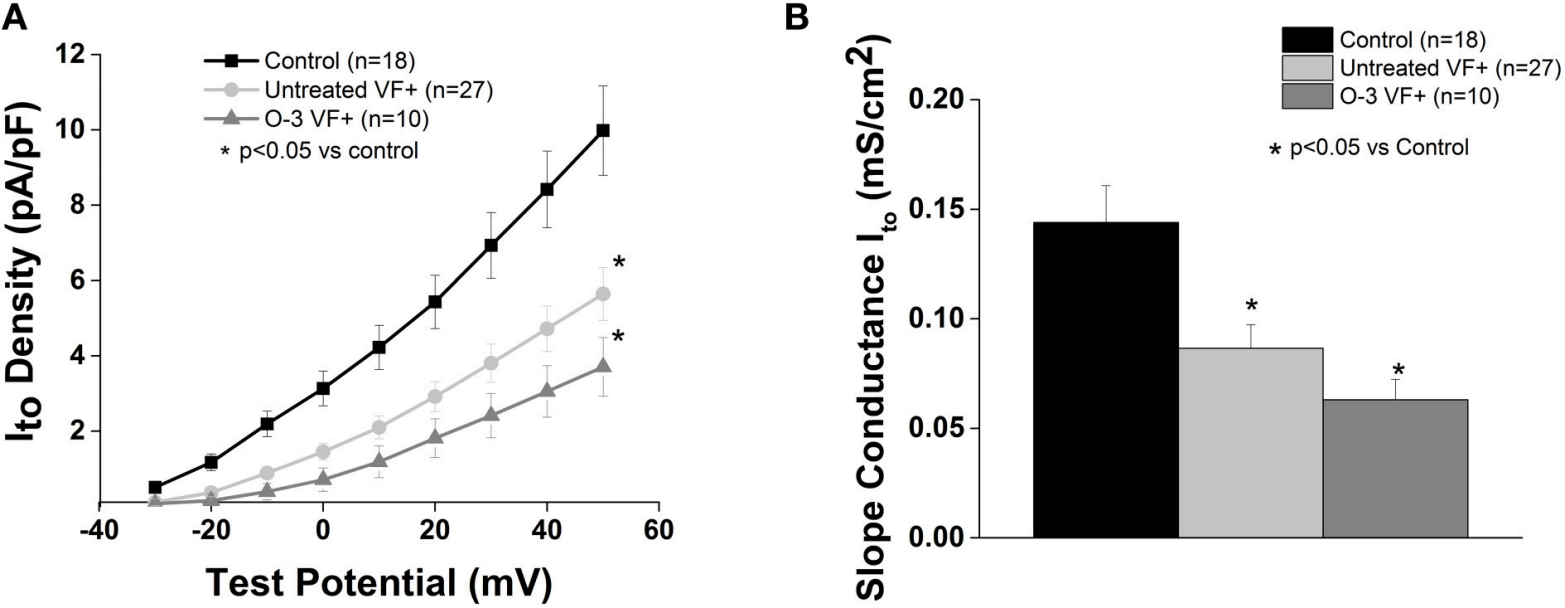
**Omega-3 PUFA treatment does not affect I_to_ in VF+ myocytes. (A)** I–V curve and **(B)** Slope conductance of control, untreated VF+, and omega-3 PUFA treated VF+ cardiomyocytes. I_to_ is reduced in VF+ myocytes and unaffected by omega-3 PUFA treatment (*P* < 0.05 vs. control). Three to seven animals per group; *n* = number of cells.

Consistent with our previous report (Sridhar et al., 2008), I_to_ was reduced in myocytes from VF+ canines (*p* < 0.5 vs. control; Figure 5). Omega-3 PUFA treatment had no effect on I_to_ vs. untreated VF+ canines.

### Interstitial fibrosis

Right ventricular tissue fibrosis was 14.0 ± 1.1%, and unchanged by infarction in either the VF− group (10.5 ± 1.2%) or VF+ group (10.8 ± 5.6%). Omega-3 PUFA treatment did not alter fibrosis in either the VF− or VF+ group (data not shown, *p* = NS).

## Discussion

In the present study we tested the hypothesis that dietary omega-3 PUFA supplementation would mediate electrophysiologic effects evident at the cellular level in the post-infarction setting. A major finding of this study is that in post-MI animals resistant to VF, omega-3 PUFA treatment caused a significant prolongation of the action potential and did not have beneficial effects on repolarization instability, consistent with pro-arrhythmic potential. Additionally, dietary Omega-3 PUFAs significantly shortened the action potential of dogs susceptible to VF (VF+) compared to untreated dogs, but did not consistently improve repolarization instability. Collectively, while we did observe cellular electrophysiologic effects of omega-3 PUFA treatment, these were insufficient to protect against malignant arrhythmias induced by acute myocardial ischemia *in vivo* in the VF+ animals.

Epidemiological studies from Bang and Dyerberg suggests beneficial results from the intake of omega-3 PUFAs, based on initial observations in a population of Greenland Inuits (Bang et al., 1971). This led to the hypothesis that increased omega-3 PUFA consumption is inversely correlated with SCD, although more recent data suggests genetic adaptations to dietary omega-3 PUFAs may exist in this population, and may contribute to the observed effects (Fumagalli et al., 2015). Early clinical and investigational trials confirmed this inverse relationship. For example, the GISSI-Prevenzione trial reported a 45% reduction in sudden death in post-MI patients treated with omega-3 PUFA supplements (Marchioli et al., 2002). However, recent clinical trials for the secondary prevention of adverse cardiovascular events following myocardial infarction such as the OMEGA and Alpha-Omega trial have not replicated the earlier success seen in the GISSI-Prevenzione study (Billman, 2013). In addition, animal studies have not provided a clear consensus on the pro- or anti-arrhythmic properties of omega-3 PUFAs (Billman, 2013). This may be attributed to differences in acute vs. chronic administration of omega-3 PUFAs (Billman, 2013). While studies have shown that acute application of omega-3 PUFAs reduce several cardiac ionic currents including: I_to_, I_Na_, and I_Kr_, the generalizability of these results to chronic supplementation is not clear (Xiao et al., 1995; Jude et al., 2003; Guizy et al., 2005). For example, a recent report shows that acute application of omega-3 PUFAs in COS7 cells increases Kv.7.1/KCNE1 (subunit which encodes slow delayed rectifying potassium channel I_Ks_) while chronic application modified channel activity resulting in pro-arrhythmic behavior (Moreno et al., 2015). In the present study, and in the companion *in vivo* studies (Billman et al., 2010, 2012) we sought to evaluate the role of chronic supplementation, and the consequent membrane incorporation of omega-3 PUFAs, in a canine model of SCD.

It is known that action potential prolongation in certain settings may favor the development of triggered arrhythmias (Rubart and Zipes, 2005). However, repolarization changes may be pro- or anti-arrhythmic depending on the underlying substrate (den Ruijter et al., 2007). Abnormal repolarization may occur in individual cells or at the tissue level with resulting increases in the propensity for SCD, which uncoupled cells being more susceptible to arrhythmias (Rubart and Zipes, 2005). In VF− animals, chronic omega-3 PUFA treatment resulted in pro-arrhythmic actions, both *in vivo* (Billman et al., 2012) and at the isolated myocyte level as shown in the present publication. An interesting finding of the present study is the discrepancy between modulation of APD and arrhythmic potential. Although, the VF+ myocytes did show attenuation of AP prolongation after omega-3 PUFA treatment, this was insufficient to translate to a reduced arrhythmia potential. It is possible that the magnitude of attenuated AP prolongation was insufficient to protect against malignant arrhythmias induced by acute myocardial ischemia *in vivo*. We used short term variability or beat to beat variability of APD as an *ex vivo* parameter to measure the instability of repolarization (Oosterhoff et al., 2007). Increases in short term variability of repolarization has shown to be predictive of ventricular arrhythmias in canine and human models of disease, when repolarization is compromised and early after depolarizations contribute to arrhythmogenesis—as we have previously reported occurs in the VF+ model (Oosterhoff et al., 2007; Sridhar et al., 2008). Our data suggest that increased myocyte repolarization variability may correspond to the previously observed pro-arrhythmic risk.

We have previously reported that chronic omega-3 PUFA treatment in this model results in dysregulation of intracellular calcium handling (Belevych et al., 2013), a potential contributor to cellular arrhythmias. Collectively this suggests that at the cellular level calcium dysregulation, rather than abnormal repolarization in isolation, may contribute to the lack of anti-arrhythmic efficacy of this treatment approach, while contributing to pro-arrhythmic potential in some subjects.

Cardiac transient outward potassium current I_to_ is responsible for partial repolarization during phase 1 of the cardiac action potential (Niwa and Nerbonne, 2010). We previously reported that a reduction in I_to_ alone was seen in VF− myocytes, and therefore is insufficient to increase arrhythmia susceptibility in this model (Sridhar et al., 2008). However, block of an additional repolarizing current (I_Kr_) was sufficient to induce cellular arrhythmias in VF− myocytes (Sridhar et al., 2008). This suggests a role for abnormal “repolarization reserve” as part of the substrate for arrhythmias in this post-MI model. In the present study we evaluated I_to_ with dietary omega-3 PUFA supplementation in a canine model of sudden death, and there was no effect on I_to_ in either group. This finding is consistent with our previous results (Sridhar et al., 2008) and the notion that reduced I_to_ does not play a significant role in altering lability of repolarization in canine models unless other electrophysiologic parameters are altered. It is therefore likely that other repolarizing currents are augmented by omega-3 PUFA treatment resulting in shortened APD in the VF+ myocytes. Verkerk et al. showed that chronic omega-3 PUFA treatment in male pigs resulted in increased outward I_K1_ and I_Ks_, both of which would contribute to accelerated repolarization; these currents may contribute to the accelerated repolarization we observed in the VF+ omega-3 PUFA treated group (Verkerk et al., 2006). Omega-3 PUFAs have been suggested to have possible anti-fibrotic effects (Wang and O'Horo, 2011; Eclov et al., 2015). Our data do not support an anti-fibrotic effect of omega-3 PUFA treatment, although our post-MI model does not result in significant right ventricular fibrosis and may limit the ability to detect an anti-fibrotic response.

## Limitations of the study

The American Heart Association recommends 1 g of EPA+ DHA supplementation for patients with existing CHD. Identifying dose equivalents between animals and humans remains a challenge (Billman, 2013). The 1–4 g EPA + DHA given to canines in the present study would be considered higher than the normal human equivalent for individuals with existing CHD. However, dosage used in the present study reflects previously reported RBC and cardiac tissue levels (Billman, 2012) that are associated with a reduction in sudden death in epidemiological studies (Siscovick et al., 1995; Albert et al., 2002).

This study did not comprehensively evaluate ion currents contributing to APD. Rather the integrated response to ion currents, the action potential was used to evaluate the response to chronic omega-3 PUFA treatment, within a rate range of 30–120 BPM. While these rates capture the majority of the daily heart rate range in canines, it is possible that results at higher rates, which may occur physiologically in dogs, would have different results. Isolated myocytes are by definition uncoupled and therefore may have intrinsically longer repolarization and more repolarization instability. Tissue recordings in coupled myocardium would be required to more closely reflect the cellular repolarization *in vivo*. Another consideration for interpreting the *in vivo* effects of omega-3 PUFAs is the potential for modulation of connexin activity (Baum et al., 2012; Radosinska et al., 2013), a possibility not examined in our model.

## Conclusion

Chronic administration of omega-3 PUFAs results in variable electrophysiologic responses. Our previous *in vivo* reports in this canine model show that such treatments may be ineffective in VF+ animals or pro-arrhythmic in VF− animals (Sridhar et al., 2008; Billman, 2012; Billman et al., 2012). We previously reported that incorporated omega-3 PUFAs after chronic treatment increased the risk of developing arrhythmias by disrupting myocyte calcium handling (Belevych et al., 2013). Therefore, abnormal variations in calcium regulation in VF− myocytes could contribute to the pro-arrhythmic effects of dietary omega-3 PUFAs in a canine model of sudden death. Abnormal calcium handling in tandem with altered repolarization may contribute to the observed effects of omega-3 PUFA supplementation. Our work does not support the routine use of chronic omega-3 PUFA supplements post-MI to reduce the risk of ventricular tachy-arrhythmias.

## Author contributions

IB: data collection and analysis; wrote portions of the paper; final approval of manuscript. YN, PV, and AS: data collection and analysis; critical revision of manuscript; final approval of manuscript. SB: data analysis, wrote, and revised sections of the manuscript; final approval of manuscript. CL: data collection and analysis; final approval of manuscript. GB: designed experimental protocol, oversaw all data collection and analysis, participated in drafting manuscript; approved final version of manuscript. CC: designed experimental protocol, conducted *in vivo* studies, participated in drafting, and approved preparation of final version of manuscript.

### Conflict of interest statement

The authors declare that the research was conducted in the absence of any commercial or financial relationships that could be construed as a potential conflict of interest.
